# An Essay on Boils

**Published:** 1869-04

**Authors:** A. Job


### An Essay on Boils.

BY A. JOB.

A BOIL IS generally very small at first, and a fellow hardly notices it, but in a few days it gets to be the biggest of the two, and the chap that has it is of very little account in comparison with his boil, which then "has him." Boils appear mysteriously upon various portions of the human body, coming when and where they darn please; and often in very inconvenient places. Sometimes a solitary boil is the sum total of the affliction, but frequently there is a "rubishin' lot of em" to help the first one. If a boil comes anywhere on a person, that person always wishes it had come somewhere else, although it would puzzle him to say just where.

Some persons call them "Damboils," but such persons are addicted to profanity—the proper name is boil. If a chap has a boil he generally gets a good deal of sympathy from others—"in a horn." Whoever asks him what ails him laughs at him for his pains to answer, while many unfeeling persons make game of him, or his misfortue or boil. It is very wicked to make sport of persons with boils, they cannot help it, and often feel very bad about it. Physicians don't give boil patients much satisfaction as a general thing, although young physicians who are just beginning to practice are fond of trying their lancets on them. Boils are said to be "healthy," and judging from the way they take hold, and hang on, and ache and burn, and grow, and raise Cain generally, there is no doubt they are healthy and have good constitutions. They are generally very lively and playful at night, and it is very funny to see a chap with a good large one prospecting around his couch for a place where his boil will fit in "without hurting."

Boils tend to "purify the blood," strengthen the system, calm the nerves, restrain the profanity, tranquilize the spirits, improve the temper, and beautify the appearance. They are good things for married men who spend their evenings away from home, as they give them an opportunity to rest their night keys and get acquainted with their families. It is said that boils save the patient "a fit of sickness,' but if the sickness is not the best to have, it must be an all-fired mean thing. It is also said that a person is better after he has had them, and here is no doubt that one feels much better after having got rid of them. Many distinguished persons have enjoyed these harbingers of health. Job took the first premium at the county fair for having more achers under cultivation than any other person. Shakespeare had them and meant boils when he said, "One woe doth tread upon another's heel, so fast they follow."

There are a great many remedies for boils, most of which are well worth trying, because, if they don't do any good, they dont hurt the boils. If a chap goes down street with a boil, every man he meets will tell him of "a good thing" for it, among which are Shoemaker's wax, Mrs. Winslow's Syrup, Trix, Spalding's glue, Charlotte Russe, Gum Drops, Water-Proof Blacking, Night-Blooming Cereus, Chloroform, Kissengen, &c.

### Be Careful.

Do not remove your flannels too soon as warm weather approaches. Flannels should be worn during the entire year, in this climate, if parties are desirous of escaping colds and catarrh.—Baths should be taken twice every week and the skin thoroughly rubbed with a crash towel. The underclothing should be of fine wool and changed twice a week. Observe these precautions and you will escape the usual "colds" of Spring, and avoid a cough that will accompany you until hot weather.

### Longevity in Mexico.

It appears that aside from the many casualities which shorten human life in Mexico, other influences are favorable to a good old age. A woman recently died in the city of Mexico, aged 118, and a widow is still living there aged 120, and able to go out. Seven Indian Chiefs recently brought as prisoners to Vera Cruz, were aged respectively, 64, 68, 80, 92, 93, and 104.—Let's emigrate.

### A Clerical Surgeon.

Father Heylen, a Catholic Priest of Boom, in Belgium, performed the Caesarian operation on a young woman in order to baptise the infant before it died. The mother appears to have been alive when the operation commenced, but both mother and child succumbed. In his defence the Priest said that he performed the operation in obedience to the direct instructions of the Archbishop.

These instructions are now to be cancelled, and the clerical surgeon tried for murder.

